# Cytokine-Based Immunotherapy for Advanced Kidney Cancer: Past Results and Future Perspectives in the Era of Molecularly Targeted Agents

**DOI:** 10.1100/tsw.2007.154

**Published:** 2007-04-09

**Authors:** Camillo Porta, Chiara Paglino, Ilaria Imarisio, Lucia Bonomi

**Affiliations:** ^1^ Medical Oncology, I.R.C.C.S. San Matteo University Hospital Foundation, piazzale C. Golgi 19, I-27100 Pavia, Italy; ^2^ Laboratory of Pre-Clinical Oncology and Developmental Therapeutics, I.R.C.C.S. San Matteo University Hospital Foundation, piazzale C. Golgi 19, I-27100 Pavia, Italy

**Keywords:** cytokines, immunotherapy, molecularly targeted drugs, kidney cancer

## Abstract

Until recently, immunotherapy has been the only therapeutic option available for patients with advanced kidney cancer, even though different choices were often made on the two sides of the Atlantic Ocean. The absence of alternatives made different immunotherapeutic approaches common practice, even with few adequate randomized studies that addressed key questions, such as the best treatment and schedule, and so on. The recent registration of the first two, molecularly targeted, agents Sorafenib and Sunitinib could (and will) render many therapeutic approaches, e.g., single-agent Interferon, obsolete. In this review, we shall cover the past achievements obtained so far with cytokine-based immunotherapy and discuss the present role of immunotherapy in the era of molecularly targeted agents. In particular, specific indications for immunotherapy are emerging (e.g., the use of Interleukin-2 in patients with high CAIX expression), while new trials are ongoing to test immunotherapy in combination with molecularly targeted agents, such as Sorafenib, Sunitinib, or Bevacizumab.

## INTRODUCTION

Renal cell carcinoma (RCC) is the most common malignancy of the kidney and accounts for approximately 3% of all adult malignancies and 2% of all deaths from neoplasms. Despite not being one of the so-called "big killers", RCC incidence and mortality have steadily increased over time[1,2], with a 126 and 37% increase in incidence and annual mortality, respectively, since the 1950s[2,3].

The only treatment with curative intent for patients diagnosed as having localized RCC is surgical removal of the tumor (nephrectomy or nephron-sparing surgery); however, 30% of patients will experience disease recurrence, while up to 30% will develop metastatic RCC. The prognosis of advanced, i.e., metastatic, patients is extremely poor, the 5-year survival rate being less than 2%[4].

Overexpression of P-glycoprotein (P-gp) encoded by the multidrug resistance 1 (MDR1) gene or multidrug resistance-associated protein (MRP) (or both), as well as decreased expression of DNA topoisomerase II, is responsible for expression of the multidrug resistance (MDR) phenotype in the vast majority of RCCs[5], so that conventional chemotherapy is largely ineffective in these tumors. RCC is also resistant to radiations, radiotherapy being only useful for palliation of tumor symptoms, e.g., in the case of bone metastases[6].

The lack of significant antitumor activity observed with chemotherapy and radiation therapy, together with the recognition of the frequent presence of several immunologic dysfunctions in RCC[7], even at diagnosis and in the absence of metastases[8], have rendered this tumor a privileged field for the development and clinical application of immunotherapy.

Indeed, until recently, immunotherapy has been considered the treatment of choice for advanced RCC patients, even though the generic term “immunotherapy” comprises a vast array of different therapeutic approaches (Table 1), too often evaluated only within small, and therefore biased, phase II studies.

**TABLE I**
**Different Immunotherapeutic Approaches for Advanced RCC**

| |
|---|
| Interferons |
| Interferon-α |
| Interferon-β |
| Interferon-γ |
| Interleukins |
| Interleukin-2 |
| Interleukin-12 |
| Interleukin-21 |
| Combinations |
| Combinations of cytokines (e.g., IL-2 and IFN-α) |
| Combinations of cytokines with cellular therapies (i.e., IL-2 and LAK cells, or IL-2 and TILs) |
| Combinations of cytokines and other immune modulators (e.g., IL-2 and histamine) |
| Circulating hematopoietic progenitor cell transplantation preceeded by reduced-intensity conditioning (“mini-allo transplantation”) |
| Vaccines |
| Tumor cell-based vaccines |
| Gene-modified tumor cell vaccines |
| Dendritic cell-based vaccines |
| Heat shock proteins-based vaccines |
| Antigenic peptides-based vaccines |

During the past 2 years, two novel, molecularly targeted drugs, i.e., Sorafenib and Sunitinib, have proved, within large randomized trials performed on a global scale, to be able to change the natural history of advanced RCC[9,10]; the results of the registrative trials of these two drugs are going to change the way we treat this tumor radically, establishing a new standard for both first- and second-line treatment.

With the worldwide commercialization of both Sorafenib and Sunitinib, and with a number of other molecularly targeted agents under active evaluation for the treatment of RCC (e.g., Bevacizumab, Temsirolimus, RAD001, etc.), the future of immunotherapy in this neoplasm is challenged.

This review will deal with the results obtained so far with the cytokine-based immunotherapy and will try to address the future of immunotherapy, alone (probably in very selected indications) as well as in combination with the new molecularly targeted drugs.

## INTERFERONS

Interferons (IFNs) are members of a superfamily of regulatory proteins produced by eukaryotic cells in response to viral infections and to several other biologic or synthetic inducers. Three IFN subtypes have been described in humans: IFN-α, -β, and -γ. Both IFN-α, produced by lymphocytes and macrophages, and IFN-β, produced by fibroblasts and mesenchymal cells, are encoded on chromosome 9, share omology, and are collectively referred to as type I; IFN-γ, produced by T-lymphocytes and, to a lesser extent, NK cells, is encoded on chromosome 12 and is dissimilar to the other two in structure, binds to a distinct cell receptor, and is referred to as type II.

The precise mechanisms supporting IFN-induced antitumor activity are still not completely known; indeed, IFNs have a broad range of biological effects (Table 2) that could potentially induce a clinically significant antitumor response *in vivo*[11,12,13,14,15,16].

**TABLE 2**
**Biological Effects of IFNs Potentially Implicated in Their Antitumor Activity**

| |
|---|
| Immune stimulatory effects |
| Stimulation of $CD8^+$ cytotoxic T lymphocytes (CTLs) |
| Up-regulation of MHC antigens |
| Stimulation of antibody-dependent cellular cytotoxicity (ADCC) |
| Regulation (positive and negative) of NK cell activity |
| Regulation of macrophage activity (IFN-γ) |
| Antiangiogenetic effects |
| Antiproliferative effects on endothelial cells and on fibroblasts |
| Direct antiproliferative effects |
| Antiproliferative effects on tumor cells (cells in $G_0$ being the most sensitive) |
| Proapoptotic effects |
| Induction of Fas expression |
| Stimulation of the Jak-Stat signaling pathway (type I IFNs) |
| Effects of cell differentiation |
| Inhibitory or stimulating effect on proliferation, depending on cell type |

### Interferon-α

Following early evidence of antitumor activity against RCC using partially purified preparations of either IFN-α or lymphoblastoid IFN, recombinant IFN-α2a and -α2b became the standard preparations used in clinical practice. Both have been extensively studied in advanced RCC patients, with doses and schedules greatly varying according to individual trial, from as low as 3 MU, to as high as 50 MU. No differences in terms of antitumor activity have ever been demonstrated according to the dose of IFN used; indeed, response rates ranged between 0 and 30%, with a median of 14.5% (13 complete and 81 partial responses out of 648 patients)[16].

The dose of IFN has been empirically divided into three categories: low (i.e., less than 5 MU), intermediate (i.e., between 5–20 MU) and high (i.e., greater than 20 MU)[17]. Two retrospective analyses compared IFN doses categorized as above[18,19]; both reported higher response rates in patients treated with intermediate doses (5–20 MU), but the second found the highest responses in patients treated with doses between 5 and 10 MU[19]. Since, differently from activity, toxicity was clearly dose dependant, a dose between 5 and 10 MU t.i.w. has since been often used. Even though clearly empirical, this assumption has been considered valid until recently, if one considers that the control arm of the recently performed trials addressing the role of Sunitinib and Temsirolimus as first-line therapy for RCC, used IFN doses of 9 and 18 MU, respectively[9,20].

Four randomized trials addressed the potential role of IFN-α in prolonging survival of advanced RCC patients. In two of them, single-agent IFN-α was compared to medroxyprogesterone acetate (MPA)[21,22], while in the other two, a combination of IFN-α and vinblastine was compared with either vinblastine alone[23] or MPA[24]. The two older studies, one comparing INF-α with MPA[21] and the other comparing INF-α plus vinblastine with MPA[24], failed to show any benefit, while the other two, one comparing INF-α plus vinblastine with vinblastine alone[23] and the other comparing INF-α alone with MPA[22], showed a small, but statistically significant survival benefit in favor of IFN-α. The results of these studies are summarized in Table 3.

**TABLE 3**
**Randomized Trials Evaluating the Effect of IFN-α on Advanced RCC Patients' Survival**

| Ref. | Treatment | No. of Patients | Response Rate | Median Survival | Survival Benefit |
|---|---|---|---|---|---|
| Steineck et al.[21] | IFN-α<br>vs.<br>MPA | 30<br><br>30 | 6%<br><br>3% | 7 months<br><br>7 months | No |
| Kriegman et al.[24] | IFN-α/VBL<br>vs.<br>MPA | 41<br><br>35 | 35%<br><br>0% | 16 months<br><br>10 months | No |
| Pyrhönen et al.[23] | IFN-α/VBL<br>vs.<br>VBL | 79<br><br>81 | 16%<br><br>2% | 17 months<br><br>10 months | Yes |
| Ritchie et al.[22] | IFN-α<br>vs.<br>MPA | 167<br><br>168 | 16%<br><br>2% | 8.5 months<br><br>6 months | Yes |

## Interferon-β and -γ

IFN-β and -γ have been less extensively evaluated. The overall response rate for IFN-β was 11% (out of 71 patients enrolled in four trials)[16], while responses for IFN-γ ranged from 0–30% in 15 trials enrolling 570 patients[16]. Despite early enthusiasm, IFN-γ yielded a response rate of only 3% within a large multicenter phase II study, enrolling 202 patients[25].

Furthermore, in a randomized trial that compared IFN-γ to placebo in 197 patients with advanced RCC, response rate was 4% for IFN-γ–treated patients and 7% for placebo-treated patients, with no significant differences in both progression-free survival (PFS), i.e., 1.9 months for both groups, and median survival, i.e., 12 months for IFN-γ and 16 months for placebo[26].

The results of both the above-referenced randomized phase II study[25] and of this phase III randomized trial[26] clearly indicate that single-agent IFN-γ has no role in the therapy of RCC.

## INTERLEUKIN-2

Even though Interleukin-2 (IL-2) was the only drug approved by the Food and Drug Administration (FDA) for the treatment of advanced RCC until 2005, a number of questions concerning IL-2–based immunotherapy remains open and the relatively small number of well-conducted, numerically consistent, phase III studies addressing these issues complicates the picture further. Besides the fact that IL-2 probably kills cancer cells with several different mechanisms[27,28] — independent of the expansion, stimulation, and activation of the immune system effector cells[29] (Table 4) — it also acts, in terms of both activity and toxicity[30], as completely different drugs according to its dose and route of administration, a behavior that is common also to other anticancer agents, e.g., 5-FU.

**TABLE 4**
**Biological Effects of IL-2 Potentially Implicated in its Antitumor Activity**

| |
|---|
| Immune stimulatory effects |
| Stimulation of $CD8^+$ cytotoxic T lymphocytes (CTLs) |
| Stimulation of NK cells |
| Stimulation of eosinophils* |
| Direct effects on cancer cells |
| Cell cycle perturbations |
| Production of cytotoxic reactive oxygen species, e.g., NO |

* At present, it is not known if the eosinophilia induced by the administration of IL-2 plays a role in the antitumor activity of this cytokine and, if any, if it is positive or negative.

This makes it extremely difficult to draw general conclusions from the few phase III studies performed to date. For example, even though a long-awaited randomized study provided evidence of the superiority of the i.v. route of administration over the s.c. route[31], many researchers noticed that the schedule used in the s.c. arm could not be considered standard. Similarly, the results of the PERCY-Quattro study, presented at the 2005 ASCO meeting[32], suggesting that IFN-α is equally active, but less toxic, than s.c. IL-2 (and therefore should be preferred to IL-2 for the treatment of advanced RCC patients of intermediate prognosis), are someway biased by the dose of IL-2 used, that made it more toxic than expected on the basis of the available literature.

### High-Dose Intravenous IL-2

High-dose i.v. bolus IL-2 received FDA approval for the treatment of patients with stage IV RCC in 1992 based on data presented on 255 patients who were entered into seven phase II clinical trials[33,34]. In these studies, patients received 600,000–720,000 IU/kg of recombinant human IL-2 by 15-min infusion every 8 h during two 5-day courses (maximum, 14 doses per course) separated by 5–9 days of rest. Stable or responding patients received a maximum of two to five courses of therapy at 8- to 12-week intervals. Objective responses were seen in 37 (15%) of the 255 patients, including 17 complete responders (CRs) (7%) and 20 partial responders (PRs) (8%).

The median duration of response was 54 months for all of the responders, 20 months for PRs, and has not been reached for CRs. The median survival was 16 months for all 255 patients. A longer (>10 years) follow-up confirmed the remarkable durability of these responses[35]; indeed, the response duration curve appears to have leveled off after the 30-month time point, with 60% of the patients who achieved a complete response remaining in remission. In addition, four partial responders who underwent surgical resection of residual disease were also long-lasting, disease-free survivors.

Therefore, the patients who achieved a complete response that lasted >30 months and those individuals with partial responses rendered disease-free following surgical resection of residual disease may actually be cured. Despite the striking results presented above, high-dose i.v. bolus IL-2 is associated with significant toxic effects and cost, and is outside the feasibility of many centers.

## Intermediate-Dose Intravenous IL-2

In the attempt to reduce high-dose IL-2–related toxicities, the French Immunotherapy Group explored the activity of intermediate-dose i.v. IL-2. The French researchers thus performed a large, phase III, randomized trial comparing intermediate-dose IL-2 administered by continuous i.v. infusion plus s.c. IFN-α, with IL-2 or IFN-α administered alone[36].

Among the 425 patients enrolled, the response rate and 1-year event-free survival were significantly higher for the combination arm, although there was no significant difference in overall survival among the three treatment arms. Notably, response rate was extremely low in the two single-agent arms (6.5 and 7.5% of patients receiving IL-2 or IFN-α alone, respectively), thus rendering the results of this study quite unuseful. Indeed, since no direct comparison between high- and intermediate-dose i.v. IL-2 is available, the exact role of the latter schedule is still to be defined.

## Low-Dose Intravenous IL-2

Low-dose i.v. IL-2 regimens (with or without IFN-α) have produced similar response rates and survival in nonrandomized phase II trials, but responses appeared to be less durable than those seen with high-dose IL-2. For example, in a series of phase II trials performed sequentially by the Cytokine Working Group, 3-year, progression-free survival was 9%, and median response duration was 53 months for patients who received high-dose IL-2 compared with 2–3% and 12 months for lower-dose IL-2 and IFN regimens[37].

The researchers of the National Cancer Institute Surgery Branch thus performed a randomized trial comparing high-dose i.v. bolus IL-2 with a low-dose i.v. bolus regimen developed by Yang et al.[38]. Following the randomization of more than 110 patients, a third arm was added involving s.c. IL-2.

Among the 306 patients assigned to either high-dose or low-dose i.v. IL-2, the response rate was significantly higher with high-dose therapy, i.e., 21 vs. 13%; furthermore, duration of responses was longer in patients receiving high-dose IL-2. Despite this, no difference in terms of overall survival was evidenced. Notably, despite toxicities that were higher in the high-dose arm, no treatment-related deaths were observed in both arms, and quality of life items were roughly similar between the two treatments.

## Subcutaneous IL-2

In the early 1990s, Jens Atzpodien was the first to propose the use of s.c. IL-2 for the treatment of kidney cancer[39,40]. Such a route of administration allowed kidney cancer patients to be treated at their own homes, caused extremely low toxicities, and yielded response rates similar to those observed with more toxic regimens, at least in phase II studies.

The above results, together with the demonstration that s.c. IL-2 doses as low as 500,000 UI/$m^2$ were able to induce significant immune system activation, rendered the s.c. route extremely popular, especially in Europe.

Indeed, several phase II trials confirmed the activity and tolerability of extremely heterogeneous, s.c. IL-2 schedules[41,42,43,44,45,46,47]. Furthermore, basing on an otherwise sound immunological rationale, many European researchers, especially in Italy, concentrated also on chronically continuing the administration of low-dose IL-2 and IFN, independent of clinical response[48].

Unfortunately, only few phase III trials were performed to assess exactly the role of s.c. IL-2 for the treatment of advanced kidney cancer, in comparison to i.v. IL-2.

## Comparing i.v. with s.c. IL-2

As far as the direct comparison between the i.v. and s.c. route of administration, the already-mentioned National Cancer Institute Surgery Branch[37] trial showed only a borderline statistical significance in terms of objective responses in favor of the high-dose arm, but no differences were found in terms of overall survival.

To determine the value of outpatient s.c. IL-2 and IFN-α relative to high-dose i.v. IL-2, the Cytokine Working Group performed a prospective randomized phase III trial[31], where patients with advanced RCC were randomized to receive either outpatient IL-2 (5 MIU/m$^2$ s.c. every 8 h for three doses on day 1 then daily 5 days/week for 4 weeks) and IFN-α2b (5 MIU/m$^2$ s.c., t.i.w., for 4 weeks), every 6 weeks, or high-dose inpatient IL-2 (600,000 IU/kg per dose i.v. every 8 h, days 1–5 and 15–19; maximum, 28 doses) every 12 weeks. Tumor responses were assessed at weeks 6 and 12, then every 12 weeks. Responding patients taking IL-2 and IFN-α received up to six cycles at 6-week intervals, whereas responding patients treated with high-dose IL-2 received up to three cycles at 12-week intervals.

Among the 192 patients enrolled (96 per arm), the response rate for high-dose IL-2 was 23% (22 of 96) vs. 10% (9 of 96) for IL-2 and IFN-α ($p = 0.018$). Eight patients achieved a complete response while taking high-dose IL-2 vs. three patients taking low-dose IL-2 and IFN-α. The median response durations were 14 months for high-dose IL-2 (range, 3–50+ months) and 7 months for IL-2 and IFN-α (range, 4–38+ months; $p = 0.18$). Median overall survivals were 17 and 13 months ($p = 0.12$), favoring high-dose IL-2. Median progression-free survival was 3 months for both treatments. As far as the primary end point of the study, that was 3-year progression-free survival; nine patients taking high-dose IL-2 were progression-free at 3 years vs. two patients taking IL-2 and IFN-α ($p = 0.06$).

The benefit of high-dose i.v. IL-2 was particularly evident in patients with primary tumors in place or in those with liver or bone metastases. Furthermore, high carbonic anhydrase IX (CA IX) immunohistochemical expression in primary tumors was associated with improved overall survival and possibly response to IL-2–based therapy. In addition, all of the long-term responders to IL-2–based treatment had high CA IX expression. A subsequent, nested, case-control study confirmed that CA IX expression levels were correlated with response to IL-2, pathological risk categorization, and survival[49]. The results of these two studies are summarized in Table 5.

**TABLE 5**
**Randomized Trials of i.v. vs. s.c. IL-2 in Advanced RCC Patients**

| Ref. | Treatment Comparison | Response Rates | Survival Difference |
|---|---|---|---|
| Yang et al.[38] | High-dose i.v. IL-2<br>vs.<br>Low-dose i.v. IL-2<br>vs.<br>Low-dose s.c. IL-2 | 21%<br><br>13%<br><br>10% | *p* = n.s. |
| McDermott et al.[31] | High-dose i.v. IL-2<br>vs.<br>s.c. IL-2 and IFN-α | 23.2%<br><br>9.9% | *p* = n.s. |

An attempt to summarize the results obtained with different IL-2 schedules came from a systematic review of the available literature by Baaten et al.[50]. The scarcity of randomized phase III trials and the heterogeneity of the data made firm conclusions extremely difficult; however, it appeared that the number of complete remissions was similar between intermittent bolus injection and s.c. routes, and that these are higher than for continuous intravenous infusion schedules. The durability of the complete responses induced by intermittent bolus injection appeared superior to those induced by s.c. IL-2 and definitely higher than with continuous intravenous infusion protocols.

## SUMMARIZING THE ACTIVITY OF SYSTEMIC IMMUNOTHERAPY IN RCC: THE RESULTS OF THE COCHRANE COLLABORATION GROUP ANALYSIS

An effort to summarize the results of randomized clinical trials of cytokine-based immunotherapy as a whole in RCC was performed by the Cochrane Collaboration Group[51]. Combined data for a variety of immunotherapies gave a 12.9% overall chance of objective responses (vs. 2.5% for nonimmunotherapy approaches and 4.3% for placebo), with a median survival of only 13.3 months. The difference in remission rate between the treatments considered correlated poorly with the difference in median survival, so that response rate could not be considered a good surrogate or an intermediate outcome for survival for advanced RCC patients. Furthermore, the analysis could not identify any published randomized study of high-dose IL-2 vs. a nonimmunotherapy control, or of high-dose IL-2 vs. IFN including survival analysis, while it has been established that reduced-dose IL-2 given by intravenous bolus or by s.c. injection provides equivalent survival to high-dose IL-2, with less toxicity. The conclusions of the study were that IFN provides a modest survival benefit compared to other commonly used treatments and should be considered for the control arm for future studies of systemic agents, while IL-2 has not been validated enough within controlled randomized studies.

As in the review by Baaten[50], this comprehensive analysis clearly highlighted the difficulties of using evidence-based medicine to determine the standard of care when clinical trial data are scarce, old, retrospective, and quite heterogeneous.

## "REGIONAL" IMMUNOTHERAPY OF ADVANCED KIDNEY CANCER: IL-2 INHALATION THERAPY

Inhalation of IL-2 (given alone or associated with the s.c. administration of the same cytokine) has been reported as an active and well-tolerated tool to prevent progression of pulmonary and mediastinal metastases of advanced kidney carcinoma[52].

In particular, five-times-daily inhalation of IL-2 yielded a 21% response rate within a phase I multidose trial, while a similar multicenter phase II trial demonstrated a 29% response rate. Among 188 metastatic RCC patients treated with inhaled recombinant IL-2 at the Clinic Eppendorf in Germany, progression of pulmonary metastases was prevented in 68% of patients for a median duration of 7 months, and overall survival was significantly improved compared with expected survival (17.2 vs. 5.3 months). Furthermore, all patients, including high-risk patients, appeared to benefit[52]. Unfortunately again, no phase III trials of such a treatment schedule have been performed to date.

## IMMUNOTHERAPY IN FRONT OF MOLECULARLY TARGETED AGENTS: THE END OF THE ROAD OR AN OPPORTUNITY FOR A NEW ERA?

Does the registration of Sorafenib and Sunitinib, together with the preliminary results of other intriguing molecularly targeted agents, mean the end of the use of immunotherapy to treat advanced kidney cancer patients? Recently, a consensus panel used the RAND/University of California-Los Angeles

Appropriateness Method to evaluate systemic therapy options (as well as cytoreductive nephrectomy) in metastatic RCC, thus indirectly answering the above question[53]. After a comprehensive literature review, the expert panel rated the appropriateness of systemic options as appropriate, inappropriate, or uncertain.

IFN-α was rated as inappropriate, a marked change from past practice. This rating directly resulted from the above-cited, head-to-head studies presented at ASCO in 2006, that reported superior results with Sunitinib in patients with better risk status[10] and with Temsirolimus in poor-risk patients[20].

Low-dose IL-2 was also rated inappropriate for most patients, although there was some uncertainty regarding better-risk patients with unknown or high expression of CA IX.

Some panelists stressed the fact that high-dose i.v. IL-2 still offers the best chance for a durable response (and even for cure), albeit to a minority of patients. Furthermore, at experienced referral centers, treatment with high-dose IL-2 can be administered safely, and adverse events can be predicted and effectively managed[54] due to careful patient selection, appropriate monitoring, as well as improvement of supportive care.

Immunotherapy was generally considered inappropriate for patients who have failed other previous immunotherapy and those with poor risk status, even though combination immunotherapy was not considered explicitly by the panel.

According to this comprehensive evaluation, cytokine-based immunotherapy seems to have only a minimal role, if any, in the management of advanced RCC patients. Despite this, since the newer drugs, even though active, do not cure, there is a growing interest in the combination of immunotherapy with molecularly targeted agents[55].

Indeed, several attempts are presently under way to try to evaluate if the already-exciting results obtained to date with the above-referenced molecularly targeted agents could be improved adding cytokines to them.

At present, few conclusive data are available. As far as we are aware, only two phase II trials of such combinations have been presented to date, and both only in the form of short abstract. In the first of them[56], treatment consisted of 8-week cycles of Sorafenib (400 mg p.o. b.i.d.) + IFN-α (10 MU s.c. t.i.w.); cycles were repeated until disease progression with 2-week breaks between cycles permitted. Among the 24 patients evaluable for response (some treatment-naïve and some pretreated), the response rate by RECIST criteria was 42% (38% partial responses and 4% complete responses), while an additional 46% had stable disease for at least one cycle, including 8% with ≥20% regression. Notably, responses were seen in all metastastic sites (lungs, pleura, kidney, liver, pancreas, and nodes) and three of the 10 responses occurred in patients who had failed prior high-dose IL-2.

In the second trial[57], 67 previously untreated, advanced RCC patients received IFN-α ($10 \times 10^6$ MU s.c. t.i.w.) and Sorafenib (400 mg p.o. b.i.d.). Overall response rate (evaluated on 53 assessable patients) was 19%, with 2% complete responses and 17% partial responses.

In both trials, the combination was well tolerated, with acceptable and expected treatment-related side effects. It is noteworthy that, despite a relevant difference between the two studies, the overall response rate for the combination of Sorafenib and IFN-α was greater than expected with either IFN-α or Sorafenib alone, thus warranting further evaluation of such a schedule.

At present, both in the U.S. (Table 6) and in Europe (Table 7, referring to the trials actually ongoing in our country, i.e., Italy), several studies are ongoing, trying to assess the value of adding cytokines (mainly, IFN-α and/or IL-2) to Sorafenib, Sunitinib, or Bevacizumab.

Only in the next years shall we understand if cytokine-based immunotherapy will re-emerge from its own ash, in association with molecularly targeted agents, or if it will became just a memory of when we were not able to impact on advanced RCC patients' survival.

**TABLE 6**
**Ongoing U.S.-Based Combination Trials of Immunotherapy and Molecularly Targeted Drugs in Advanced RCC Patients, According to the NCI Trials Database***

| Study Number | Phase | Title | Objectives |
|---|---|---|---|
| A6181065 | III | “Sunitinib Malate Alone and in Combination with Interferon Alfa as First-Line Therapy for Metastatic Renal Cell Cancer” | First part: to determine the doses of two drugs that can be given safely in combination and select the doses to be given in the second part of the trial.<br>Second part, to see if the combination is any better at delaying progression of renal cell cancer than Sunitinib malate given alone in one of two dose schedules. |
| 494F01 | I/II | “Study of Recombinant Interleukin-21 in Combination with Sorafenib for Metastatic Renal Cell Carcinoma” | First part will estimate the MTD of rIL-21 given for one treatment course (consisting of two 5-day cycles of rIL-21) in combination with a standard dose of Sorafenib administered during a 6-week treatment course.<br>Second part will further evaluate the safety and preliminary antitumor activity of rIL-21 at the dose recommended from phase I in combination with Sorafenib. |
| DUMC-6258-04-9R0 | II | “Sorafenib and Interferon Alfa in Treating Patients with Locally Advanced or Metastatic Kidney Cancer” | This phase II trial is studying how well giving Sorafenib with IFN-$\alpha$ works in treating patients with locally advanced or metastatic kidney cancer. |
| MCC-13921 | II | “Bevacizumab and Interleukin-2 in Treating Patients with Metastatic Kidney Cancer” | This phase II trial is studying how well giving Bevacizumab together with IL-2 works in treating patients with metastatic kidney cancer. |
| MDA-2004-0526 | IIR** | “Sorafenib with or without Interferon Alfa-2b in Treating Patients With Metastatic Kidney Cancer” | This randomized phase II trial is studying Sorafenib and IFN-$\alpha$ to see how well they work compared to Sorafenib alone in treating patients with metastatic kidney cancer. |
| UCLA-050658-01 | II | “Bevacizumab and Interleukin-2 in Treating Patients with Metastatic Kidney Cancer” | This phase II trial is studying how well giving Bevacizumab together with IL-2 works in treating patients with metastatic kidney cancer. |

* http://www.cancer.gov/search/ResultsClinicalTrialsAdvanced.aspx?protocolsearchid=2932863.

** IIR, randomized phase II study.

**TABLE 7**
**Ongoing Italian Combination Trials of Immunotherapy and Molecularly Targeted Drugs in Advanced RCC Patients**

| Study | Phase | Title | Objectives |
|---|---|---|---|
| GOIRC 0681 RAPSODY | IIR* | "Randomized Prospective Trial of Two Schedules of Sorafenib 800 mg Daily and Interferon-Alpha in Metastatic Renal Cell Carcinoma (MRCC): A GOIRC Phase II Study" | This randomized phase II trial is studying Sorafenib and two different doses of IFN-$\alpha$ to see how well they work compared to Sorafenib alone in treating naïve patients with metastatic kidney cancer. |
| INT 38/06 ROSORC | II | "A Randomised, Open Label, Multicenter Phase II Study of First Line Therapy with Sorafenib in Association with Interleukin-2 versus Sorafenib Alone in Patients with unresectable and/or Metastatic Renal Cell Carcinoma (RCC)" | This phase II trial is studying how well giving Sorafenib together with s.c. IL-2 works in treating naïve patients with metastatic kidney cancer. |

* IIR, randomized phase II study.

## References

1. Chow, W.H., Devesa, S.S., Warren, J.L., and Fraumeni, J.F., Jr. (1999) Rising incidence of renal cell cancer in the United States. *JAMA* **281,** 1628–1631.
2. Pantuck, A.J., Zisman, A., and Belldegrun, A.S. (2001) The changing natural history of renal cell carcinoma. *J. Urol*. **166,** 1611–1623.
3. Jemal, A., Murray, T., Ward, E., et al. (2005) Cancer statistics, 2005. *CA Cancer J. Clin*. **55,** 10–30.
4. Goodley, P.A. and Taylor, M. (2001) Renal cell carcinoma. Curr. Opin. Oncol. **13**, 199–203.
5. Naito, S., Koga, H., Yokomizo, A., et al. (2000) Molecular analysis of mechanisms regulating drug sensitivity and the development of new chemotherapy strategies for genitourinary carcinomas. *World J. Surg*. **24**, 1183–1186.
6. Motzer, R.J. and Russo, P. (2000) Systemic therapy for renal cell carcinoma. *J. Urol*. **163**, 408–417.
7. Enquist, E., Zambiano, N., Zbar, B., et al. (2000) Molecular mechanisms of immune dysfunction in renal cell carcinoma. In *Renal Cell Carcinoma. Molecular Biology, Immunology and Clinical Management.* Bukowski, R.M. and Novick, A.C., Eds. Humana Press, Totowa, NJ. pp. 63–78.
8. Porta, C., Bonomi, L., and Lillaz, B., et al. (2007) Renal cell carcinoma-induced immunosuppression: an immunophenotypic study of lymphocyte subpopulations and of circulating dendritic cells in patients at first diagnosis. *Anticancer Res.,* in press.
9. Escudier, B., Eisen, T., and Stadler, W.M., et al. (2007) Sorafenib in advanced clear-cell renal-cell carcinoma. *N. Engl. J. Med.* **356,** 185-187.
10. Motzer, R.J., Hutson, T.E., and Tomczak, P., et al. (2007) Sunitinib versus Interferon alfa in metastatic renal-cell carcinoma. *N. Engl. J. Med.* **356**, 115-124.
11. Tagliaferri, P., Caraglia, M., Budillon, A., et al. (2005) New pharmacokinetic and pharmacodynamic tools for interferon-alpha (IFN-alpha) treatment of human cancer. *Cancer Immunol. Immunother.* **54,** 1–10.
12. Pokrovskaja, K., Panaretakis, T., and Grander, D. (2005) Alternative signaling pathways regulating type I interferon-induced apoptosis. *J. Interferon Cytokine Res*. **25**, 799–810.
13. Caraglia, M., Marra, M., Pelaia, G., et al. (2005) Alpha-interferon and its effects on signal transduction pathways. *J. Cell Physiol.* **202**, 323–335.
14. Parmar, S. and Platanias, L.C. (2003) Interferons: mechanisms of action and clinical applications. *Curr. Opin. Oncol.* **15**, 431–439.
15. Clemens, M.J. (2003) Interferons and apoptosis. *J. Interferon Cytokine Res*. **23,** 277–292.
16. Motzer, R.J. and Berg, W.J. (2000) Role of Interferon in metastatic renal cell carcinoma. In *Renal Cell Carcinoma. Molecular Biology, Immunology and Clinical Management.* Bukowski, R.M. and Novick, A.C., Eds. Humana Press, Totowa, NJ. pp. 319–329.
17. Krown, S.E. (1987) Interferon treatment of renal cell carcinoma: current status and future prospects. *Cancer* **59**, 647–651.
18. Wirth, M.P. (1993) Immunotherapy for metastatic renal cell carcinoma. *Urol. Clin. North Am.* **20,** 283–295.

19. Savane, P.D. and Muss, H.B. (1995) Renal cell cancer. In *Biological Therapy*. De Vita, V.T., Hellman, S., and Rosenberg, S.A., Eds. JB Lippincott, Philadelphia. pp. 373–387.
20. Hudes, G., Carducci, M., Tomczak, P., et al. (2006) A phase 3, randomized, 3-arm study of temsirolimus (TEMSR) or interferon-alpha (IFN) or the combination of TEMSR + IFN in the treatment of first-line, poor-risk patients with advanced renal cell carcinoma (adv RCC). *Abstr. Am. Soc. Clin. Oncol.* **24(18S),** LBA4.
21. Steineck, G., Strander, H., Carbin, B.E., et al. (1990) Recombinant leukocyte interferon alpha-2a and medroxyprogesterone in advanced renal cell carcinoma. A randomized trial. *Acta Oncol.* **29,** 155–162.
22. Ritchie, A.W.S., Griffiths, G., Cook, P., et al. (1998) Alpha interferon improves survival in patients with metastatic renal cell carcinoma. Preliminary results of an MRC randomized trial. *Proc. Am. Assoc. Clin. Oncol*. **17**, 310a.
23. Pyrhönen, S., Salminen, E., Lehtonen, T., et al. (1996) Recombinant interferon alpha-2a with vinblastine vd. vinblastine alone in advanced renal cell carcinoma. A phase III study. *Proc. Am. Assoc. Clin. Oncol.* **15,** 244a.
24. Kriegmar, M., Oberneder, R., and Hofstetter, A. (1998) Interferon alpha and vinblastine versus medroxy-progesterone acetate in the treatment of metastatic renal cell carcinoma. *Urology* **45,** 758–762.
25. Small, E.J., Weiss, G.R., Malik, U.K., et al. (1998) The treatment of metastatic renal cell carcinoma patients with recombinant human gamma interferon. *Cancer J. Sci. Am.* **4**, 162–167.
26. Gleave, E.M., Elhilali, M., Fradet, Y., et al. (1998) Interferon gamma-1b compared with placebo in metastatic renal cell carcinoma. *N. Engl. J. Med*. **338,** 1265–1271.
27. Porta, C., Danova, M., Orengo, A.M., et al. (2000) Interleukin-2 induces cell cycle perturbations leading to cell growth inhibition and death in malignant mesothelioma cells *in vitro*. *J. Cell Physiol.* **185,** 126–134.
28. Porta, C., Rizzo, V., Zimatore, M., et al. (2002) Intrapleural interleukin-2 induces nitric oxide production in pleural effusions from malignant mesothelioma: a possible mechanism of interleukin-2-mediated cytotoxicity? *Lung Cancer* **38**, 159–162.
29. Olencki, T. and Bukowski, R.M. (2000) Interleukin-2 in metastatic renal cell carcinoma. In *Renal Cell Carcinoma. Molecular Biology, Immunology and Clinical Management.* Bukowski, R.M. and Novick, A.C., Eds. Humana Press, Totowa, NJ. pp. 301–318.
30. Porta, C., Moroni, M., Bobbio-Pallavicini, E., Tinelli, C., and Regazzi-Bonora, M. (1997) Nitrate plasma level as a marker of nitric oxide production after subcutaneous interleukin 2 immunotherapy. *J. Natl. Cancer Inst*. **89,** 1545.
31. McDermott, D.F., Regan, M.M., Clark, J.I., et al. (2005) Randomized phase III trial of high-dose interleukin-2 versus subcutaneous interleukin-2 and interferon in patients with metastatic renal cell carcinoma. *J. Clin. Oncol*. **23,** 133–141.
32. Negrier, S., Perol, D., Ravaud, A., et al. (2005) Do cytokines improve survival in patients with metastatic renal cell carcinoma (MRCC) of intermediate prognosis? Results of the prospective randomized PERCY-Quattro trial. *Abstr. Am. Soc. Clin. Oncol*. **23,** 380s (Abstr. LBA4511).
33. Fyfe, G., Fisher, R.I., Rosenberg, S.A., et al. (1995) Results of treatment of 255 patients with metastatic renal cell carcinoma who received high-dose recombinant interleukin-2 therapy. J. *Clin. Oncol.* **13,** 688–696.
34. Fisher, R.I., Rosenberg, S.A., and Fyfe, G. (2000) Long-term survival update for high-dose recombinant interleukin-2 in patients with renal cell carcinoma. *Cancer J. Sci. Am*. **6(Suppl 1),** S55–57.
35. McDermott, D.F. and Atkins, M.B. (2004) Application of IL-2 and other cytokines in renal cancer. *Expert Opin. Biol. Ther*. **4**, 455–468.
36. Negrier, S., Escudier, B., Lasset, C., et al. (1998) Recombinant human interleukin-2, recombinant human interferon alpha-2a, or both in metastatic renal cell carcinoma. Groupe Francais d'Immunotherapie. *N. Engl. J. Med*. **338**, 1272–1278.
37. McDermott, D.F., Regan, M.M., and Atkins, M.B. (2006) Interleukin-2 therapy of metastatic renal cell carcinoma: update of phase III trials. *Clin. Genitourinary Cancer* **5**, 114–119.
38. Yang, J.C., Sherry, R.M., Stienberg, S.M., et al. (2003) A three-arm randomized comparison of high and low dose intravenous and subcutaneous interleukin-2 in the treatment of metastatic renal cancer. *J. Clin. Oncol*. **21,** 3127–3132.
39. Atzpodien, J., Körfer, A., Palmer, P.A., et al. (1990) Treatment of metastatic renal cell cancer patients with recombinant subcutaneous human interleukin-2 and interferon-alpha. *Ann. Oncol.* **1,** 377–378.
40. Kirchner, H., Körfer, A., Palmer, P.A., et al. (1990) Subcutaneous interleukin-2 and interferon-alpha 2b in patients with metastatic renal cell cancer: the German outpatient experience. *Mol. Biother.* **2,** 145–154.
41. Sleijfer, D.T., Janssen, R.A., Buter, J., et al. (1992) Phase II study of subcutaneous interleukin-2 in unselected patients with advanced renal cell cancer on an outpatient basis. *J. Clin. Oncol*. **10**, 1119–1123.
42. Vogelzang, N.J., Lipton, A., and Figlin, R.A. (1993) Subcutaneous interleukin-2 plus interferon alfa-2a in metastatic renal cancer: an outpatient multicenter trial. *J. Clin. Oncol*. **11**, 1809–1816.
43. Ravaud, A., Negrier, S., Cany, L., et al. (1994) Subcutaneous low-dose recombinant interleukin 2 and alpha-interferon in patients with metastatic renal cell carcinoma. *Br. J. Cancer* **69**, 1111–1114.
44. Facendola, G., Locatelli, M.C., Pizzocaro, G., et al. (1995) Subcutaneous administration of interleukin 2 and interferon-alpha-2b in advanced renal cell carcinoma: a confirmatory study. *Br. J. Cancer* **72**, 1531–1535.
45. Canobbio, L., Curotto, A., Cannata, D., et al. (1996) Combination therapy with subcutaneous interleukin-2 and interferon-alpha in advanced renal cancer patients with poor prognostic factors. *Anticancer Res*. **16**, 541–544.
46. Tourani, J.M., Lucas, V., Mayeur, D., et al. (1996) Subcutaneous recombinant interleukin-2 (rIL-2) in out-patients with metastatic renal cell carcinoma. Results of a multicenter SCAPP1 trial. *Ann. Oncol.* **7,** 525–528.

47. Buzio, C., Andrulli, S., Santi, R., et al. (2001) Long-term immunotherapy with low-dose interleukin-2 and interferon-alpha in the treatment of patients with advanced renal cell carcinoma. *Cancer* **92,** 2286–2296.
48. Porta, C. (2006) Maintenance biotherapy with interleukin-2 and interferon for metastatic renal cell cancer. *Expert Rev. Anticancer Ther.* **6,** 141–152.
49. Atkins, M., McDermott, D., Mier, J., et al. (2004) Carbonic anhydrase IX (CAIX) expression predicts for renal cell cancer (RCC) patient response and survival to IL-2 therapy. *Proc. Am. Soc. Clin. Oncol*. **22(14S),** 4512.
50. Baaten, G., Voogd, A.C., and Wagstaff, J. (2004) A systematic review of the relation between interleukin-2 schedule and outcome in patients with metastatic renal cell cancer. *Eur. J. Cancer* **40,** 1127–1144.
51. Coppin, C., Porzsolt, F., Kumpf, J., Coldman, A., and Wilt, T. (2000) Immunotherapy for advanced renal cell cancer. *Cochrane Database Syst. Rev*. **3,** CD001425.
52. Huland, E., Heinzer, H., Huland, H., and Yung, R. (2000) Overview of interleukin-2 inhalation therapy. *Cancer J. Sci. Am.* **6(Suppl 1),** S104–112.
53. Halbert, R.J., Figlin, R.A., Atkins, M.B., et al. (2006) Treatment of patients with metastatic renal cell cancer: a RAND Appropriateness Panel. *Cancer* **107,** 2375–2383.
54. Schwartzentruber, D.J. (2001) Guidelines for the safe administration of high-dose interleukin-2. *J. Immunol.* **24,** 287–293.
55. Reddy, G.K. and Bukowski, R.M. (2006) Sorafenib: recent updates on activity as single agent and in combination with interferon-alpha2 in patients with advanced stage renal cell carcinoma. *Clin. Genitourinary Cancer* **4,** 246–248.
56. Gollob, J., Richmond, T., Jones, J., et al. (2006) Phase II trial of sorafenib plus interferon-alpha 2b (IFN-α2b) as first- or second-line therapy in patients (pts) with metastatic renal cell cancer (RCC). *Proc. Am. Assoc. Clin. Oncol*. **24(18S),** 4538A.
57. Ryan, C.W., Goldman, B.H., Lara, P.N., et al. (2006) Sorafenib plus interferon-□2b (IFN) as first-line therapy for advanced renal cell carcinoma (RCC): SWOG 0412. *Proc. Am. Assoc. Clin. Oncol.* **24(18S),** 4525A.
